# Supplementary material for: Coherent coupling of molecular resonators with a microcavity mode
Source: Nat Commun. 2015 Jan 13;6:5981. doi: 10.1038/ncomms6981 (PMC4308833; doi:10.1038/ncomms6981)
Supplement: Supplementary Information — Supplementary Figure 1, Supplementary Table 1, Supplementary Notes 1-4, and Supplementary References [file ncomms6981-s1.pdf]

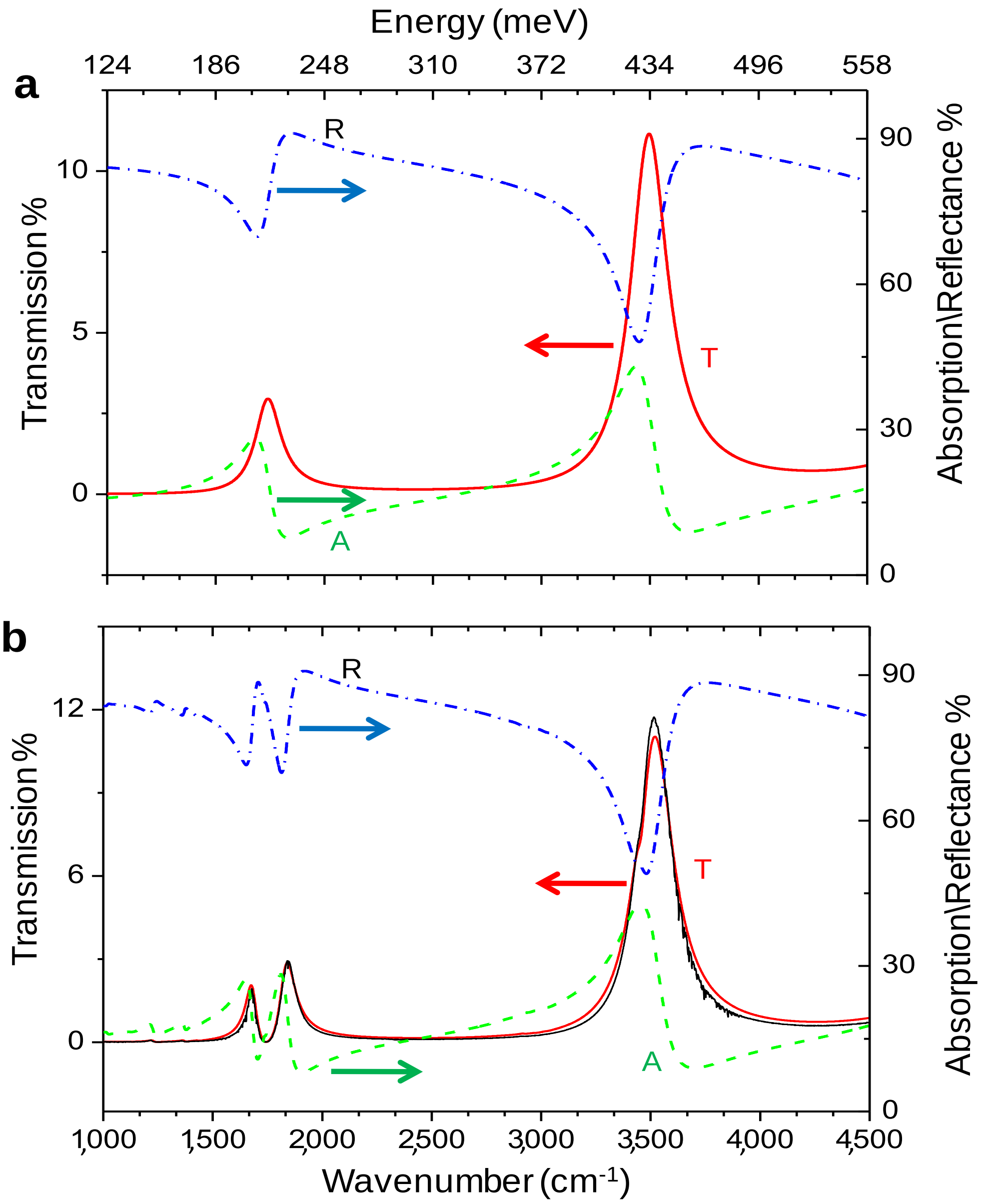

Supplementary Figure 1| Transmission, absorption, and reflection of the un-coupled and coupled cavity. **a**, Transmission (red solid line), absorption (green dashed line), and reflection (blue dashed dotted line) of the uncoupled cavity using the best fit parameters from the measured transmission spectrum. **b**, Transmission (red solid line) with the measured spectrum (black solid line), absorption (green dashed line), and reflection (blue dashed dotted line) of the coupled cavity.

ω_p	f_0	Γ_0*	f_1	Γ_1	ω_1	f_2	Γ_2	ω_2	f_3	Γ_3	ω_3	f_4	Γ_4	ω_4	f_5	Γ_5	ω_5
9.03	0.76	0.05	0.02	0.24	0.41	0.01	0.34	0.83	0.07	0.870	2.96	0.60	2.49	4.30	4.38	2.21	13.32

Supplementary Table 1 Gold dielectric constant parameters according to Eq. 2, all the parameters are given in eV units. * The total damping term was considered as 0.125 in the simulations.

Supplementary Note 1

Cavity preparation and dispersion measurements

An approximately 10 nm- thick Au mirror was sputtered on a clean Ge substrate. Then, a polymer (Poly Vinyl Acetate or PVAc) film was deposited by spin-casting (4230 rpm) to form a layer of about 2 microns thickness tuned to overlap the first cavity mode with the (C=O) bond stretching band of PVAc at 1740 cm^{-1}.

PVAc (MW: 140000) was dissolved in Toluene (15 wt. %) and mechanically steered at 100 °C for 40 hours, cooled to room temperature and passed thru a 0.22 μm Nylon filter prior to spin-casting. Finally, the cavity was formed by sputtering a second Au layer (10 nm) directly on top of the polymer. The thickness of the Au mirrors was optimized to obtain sufficient intensity in the transmission spectra in the required spectral region. Due to the significant increase of both the real and imaginary parts of the dielectric constant of Au in the infra-red (IR) region, a compromise was necessary between the cavity quality factor and the transmission mode intensity.

To measure the net polymer absorption without the cavity, another sample of the same thickness was prepared by spin-casting the PVAc solution directly on top of a clean Ge substrate.

The spectra of the cavity were acquired by standard FTIR (Fourier transform infra-red) spectrometer (Nicolet 6700) in transmission mode (description of the measurement set-up is given in Figure 2c in the main text). Prior to every measurement, a background was acquired in order to normalize the actual measurement and avoid baseline instability. All the measurements were performed with a

resolution of 1 cm^{-1} and averaged over 128 scans to enhance the signal to noise (SNR) in the spectral range 400-7400 cm^{-1}.

The dispersion of the two hybrid states of the coupled system was measured by varying the incidence angle in the range from -60° to +60° (as shown in Figure 3 in the main text). The position of the bare cavity mode was tuned by varying the in-plane wave vector k_x with the incidence angle ($k = 10^4/(2nd\cos\theta_i)$) whereas k is the first cavity mode wave-vector in cm^{-1}, n the background polymer refractive index, d the cavity thickness, and θ_i the incidence angle.

Supplementary Note 2

Theoretical fit of the transmission spectra

In order to simulate the optical response of the polymer in the IR region, the Lorenz model was used to describe the molecular polarizability. Since the absorption of the uncoupled polymer was obtained from the transmission of a bare polymer thin film on Ge substrate, an interference pattern was observed with values higher than 100% (negative absorbance values). This interference effect is observed when the measurement is normalized to the substrate transmission. In order to estimate the real absorption of the bare film, the Lorenz model with multiple resonances in the relevant spectral range was assumed as follows[1]:

$$\varepsilon(k) = \varepsilon_B - \sum_{j=1}^{N} \frac{f_j}{k^2 - k_{0j}^2 + ik\Gamma_j} \qquad (1)$$

Here, $\varepsilon_{B,}$ f_j, k_{0j}, and Γ_j are respectively the background dielectric contribution, oscillator strength, resonance wave vector, and the phenomenological damping constant of vibrational band *j*. The absorption intensity of the band is determined by both f_j and Γ_j whereas the FWHM is

solely governed by Γ_j . In our fit procedure, all these parameters together with the thin film thickness were varied to obtain the best fit with the experimental measurements. The best fit parameters for the (C=O) bond absorption band were $f_j = 50\times10^3$, $k_{0j} = 1739 cm^{-1}$, $\Gamma_j = 13 cm^{-1}$ and the background refractive index (RI) is $n_B = \sqrt{\varepsilon_B} = 1.41$.

The dispersion of the Au mirrors, on the other hand, was modeled by the Lorenz-Drude equation (2). In this model, the contributions of the intra-band and the inter-band transitions are explicitly separated as can be seen in Eq. (2)[2]:

$$\varepsilon_m = \varepsilon_{free-electrons} + \varepsilon_{bound-electrons}$$

$$\varepsilon_{free-electrons} = 1 - \frac{f_0\omega_p^2}{\omega(\omega - i\Gamma_0)} \quad ; \quad \varepsilon_{bound-electrons} = \sum_{j=1}^{K} \frac{f_j\omega_p^2}{(\omega_j^2 - \omega^2) + i\omega\Gamma_j} \qquad (2)$$

In the last two terms, ω_p is the plasma frequency, K is the number of oscillators involved, each with frequency ω_j , strength f_j and life time $1/\Gamma_j$.

Since the thickness of the Au mirrors used in our study is very small, the dielectric constant becomes size dependent. For noble metals, this applies to structures smaller than the conduction electron mean-free path which is roughly 20 nm or less in the smallest dimensions[3]. The most significant influence of the size-dependence on the dielectric function is broadening of the plasmon width due to electron scattering at the boundaries. In order to describe this effect, it is convenient to consider the same Lorenz-Drude model as in Eq. (2) with the introduction of additional damping term Γ_S to the normal damping Γ_0 which is proportional to v_F/L where v_F and L are the Fermi velocity and the mirror thickness respectively. Within our theoretical procedure, this additional term was varied to improve the fit with the experimental measurements. The parameters of Au were adopted from Rakic *et al.* (see Supplementary Table 1)[2]. The overall damping term $\Gamma_{tot} = \Gamma_0 + \Gamma_S$ which gave the best fit with the experimental results was found to be $2.5\Gamma_0$.

The transmission of the entire cavity was calculated using the standard 2x2 propagation matrices for a multilayer system embedded between two semi-infinite dielectric, isotropic, and homogenous media[4]. Correction for the transmission from the rear Ge/air interface was performed using the transmission spectrum of the bare Ge substrate.

After retrieving the optimal parameters of the cavity, the dispersion for the empty cavity was estimated by eliminating the absorption band's contributions from the dielectric constant. The splitting in reflection and absorption are compared to the transmission measurements as shown in Supplementary Figure 1. The observed values of transmission as well as the spectral shapes are in excellent agreement with the theoretical calculations. However, from the reflection and absorption spectra, one can see that the splitting is dependent on the measurement type as was confirmed in our previous works[5]. The observed splitting in transmission (Ω_T) is 167 cm^{-1} (20.7 meV), whereas the splitting in absorption (Ω_A) and reflection (Ω_R) were slightly smaller (162 $cm^{-1}$and 161 cm^{-1} respectively). The ratio (Ω_T / Ω_A) is very close to unity which is an indication of high coupling strength[5].

In the measured spectra, one can note some asymmetries between the LP and UP spectra at normal incidence (Figure 3a in the main text). The small spectral asymmetry of the two hybrid states with respect to the vibrational band frequency is mainly caused by the background effect of the polymer. We assumed for the uncoupled cavity that the background polarizability is frequency independent. In fact, the non-uniform free spectral range (FSR), measured for higher modes of control cavities made on glass substrates, showed that the background slightly disperses through the spectral range. The intensities and shapes of the polariton profiles, on the other hand, are mainly affected by the dispersion of the metallic mirrors as well as the vibrational band profile. From the excellent fit of the measured spectra (Figure 4d), we can ascertain the difference in polariton peak intensities to the dispersive response of the metal. Finally, added to the dispersion of the metal, the original

asymmetry in the (C=O) bond vibrational mode explains the differences in the shapes of the two polariton spectral signatures.

Supplementary Note 3

Field distribution

The electromagnetic field intensity calculations were based on a general algorithm considering the well known 4x4 propagation matrices in stratified media. In our case, the layers constituting the cavity, namely the two thin Au mirrors and the polymer layer were assumed to be isotropic and homogeneous and the incident field to be arbitrary polarized. In all the calculations, the experimental beam divergence and the approximately Gaussian beam shape of the FTIR were taken into account.

Supplementary Note 4

Estimation to the number of (C=O) bonds inside the cavity volume

The (C=O) density in the cavity can be calculated by dividing the PVAc density (1.18 g/cm^3) by the PVAc monomer weight (86.09 g/mole) and multiplying by Avogadro's number, yielding 8.25x10^{21} (C=O) per cm^3. This implies that in the mode volume of the cavity (ca. 10^{-4} cm^3) there are about 8.25x10^{17} (C=0) bonds.

Supplementary References

1. Oughstun, K. E. & Cartwright, N. A. On the Lorentz-Lorenz formula and the Lorenz model of dielectric dispersion. *Opt. Express* **11,** 13, 1541-1546 (2003).

2. Rakic, A. D. , Djurišic, A. B. , Elazar, J. M. & Majewski, M. L. Optical properties of metallic films for vertical-cavity optoelectronic devices. *Appl. Opt.* **37,** 22, 5271-5283 (1998).

3. Kreibig, U. Electronic properties of small silver particles: the optical constants and their temperature dependence. *J. Phys. F: Metal Phys.* **4,** 999-1014 (1998).

3. Born, M. & Wolf, E. *Principles of Optics: Electromagnetic Theory of Propagation, Interference and Diffraction of Light.* (7th Ed., Cambridge press, 1999)

5. Schwartz, T., Hutchison, J. A., Genet, C. & Ebbesen, T. W. Reversible switching of ultrastrong light-molecule coupling. *Phys. Rev. Lett.* **106,** 196405-196408 (2011).
